# Identification of embryonic lethal genes in humans by autozygosity mapping and exome sequencing in consanguineous families

**DOI:** 10.1186/s13059-015-0681-6

**Published:** 2015-06-03

**Authors:** Hanan E. Shamseldin, Maha Tulbah, Wesam Kurdi, Maha Nemer, Nada Alsahan, Elham Al Mardawi, Ola Khalifa, Amal Hashem, Ahmed Kurdi, Zainab Babay, Dalal K. Bubshait, Niema Ibrahim, Firdous Abdulwahab, Zuhair Rahbeeni, Mais Hashem, Fowzan S. Alkuraya

**Affiliations:** Department of Genetics, King Faisal Specialist Hospital and Research Center, Riyadh, Saudi Arabia; Department of Obstetrics and Gynecology, King Faisal Specialist Hospital and Research Center, Riyadh, Saudi Arabia; Department of Obstetrics and Gynecology, Security Forces Hospital, Riyadh, Saudi Arabia; Department of Medical Genetics, King Faisal Specialist Hospital and Research Center, Riyadh, Saudi Arabia; Department of Pediatrics, Prince Sultan Military Medical City, Riyadh, Saudi Arabia; Department of Obstetrics and Gynecology, Prince Sultan Military Medical City, Riyadh, Saudi Arabia; Department of Obstetrics and Gynecology, College of Medicine, King Saud University, Riyadh, Saudi Arabia; Department of Pediatrics, Faculty of Medicine, Ain Shams University, Cairo, Egypt; Department of Pediatrics, King Fahd Hospital of the University, University of Dammam, Dammam, Saudi Arabia; Department of Anatomy and Cell Biology, College of Medicine, Alfaisal University, Riyadh, Saudi Arabia

## Abstract

**Background:**

Identifying genetic variants that lead to discernible phenotypes is the core of Mendelian genetics. An approach that considers embryonic lethality as a bona fide Mendelian phenotype has the potential to reveal novel genetic causes, which will further our understanding of early human development at a molecular level. Consanguineous families in which embryonic lethality segregates as a recessive Mendelian phenotype offer a unique opportunity for high throughput novel gene discovery as has been established for other recessive postnatal phenotypes.

**Results:**

We have studied 24 eligible families using autozygosity mapping and whole-exome sequencing. In addition to revealing mutations in genes previously linked to embryonic lethality in severe cases, our approach revealed seven novel candidate genes (*THSD1*, *PIGC*, *UBN1*, *MYOM1*, *DNAH14*, *GALNT14*, and *FZD6*). A founder mutation in one of these genes, *THSD1*, which has been linked to vascular permeability, accounted for embryonic lethality in three of the study families. Unlike the other six candidate genes, we were able to identify a second mutation in *THSD1* in a family with a less severe phenotype consisting of hydrops fetalis and persistent postnatal edema, which provides further support for the proposed link between this gene and embryonic lethality.

**Conclusions:**

Our study represents an important step towards the systematic analysis of “embryonic lethal genes” in humans.

**Electronic supplementary material:**

The online version of this article (doi:10.1186/s13059-015-0681-6) contains supplementary material, which is available to authorized users.

## Background

The in utero development of humans from a single-celled zygote to a newborn with trillions of remarkably diverse cells with three-dimensional organization as organs is a highly complex process that remains incompletely understood. Much of our understanding of the molecular control of human development comes from the discipline of developmental genetics. The use of animal models as surrogates to understand these molecular events by virtue of forward and reverse genetics approaches that deconvolute these complex events to the level of individual genes has been helpful but does not obviate the need to study human embryos.

In humans, the ability to trace certain birth defects to individual genetic lesions has served as a “shortcut” to accelerate the discovery of the genetic control of many aspects of normal development. The premise of this approach is that genes linked to birth defects must be indispensable to the normal development of the respective organ(s), especially in the Mendelian context where the link is directly causal. In fact, it can be argued that the study of Mendelian forms of birth defects has provided the most compelling list of genes that are required for normal human development [1].

Embryonic lethality is a well-studied phenomenon in many model organisms, and the proportion of genes that are indispensable to development is remarkable. For example, 19 % of yeast genes are lethal when knocked out [2]. A similar systematic survey has been more challenging to conduct in higher organisms but available data suggest a lethal knockout phenotype for 30 % of mouse genes in the homozygous state [3]. Since systematic knockout of each human gene to test its viability cannot be done experimentally, naturally occurring mutations that effectively knock out the respective genes provide a practical alternative. Building on the successful approach of using Mendelian genetics of birth defects to understand normal morphogenesis of individual organs, we reckoned that Mendelian forms of embryonic lethality offer a window into the essential genetic components of early organismal development in humans. In this study, we report our genomic analysis of multiplex consanguineous pedigrees in which embryonic lethality appears to follow a Mendelian recessive pattern. This approach revealed known disease genes that have been reported to cause embryonic lethality in severe cases, in addition to genes that have not been previously linked to human diseases, representing novel candidates. The candidacy of one of these novel genes is further supported by positional mapping data as well as the identification of two different mutations.

## Results

### Identification of Mendelian forms of embryonic lethality

**Fig. 1:**
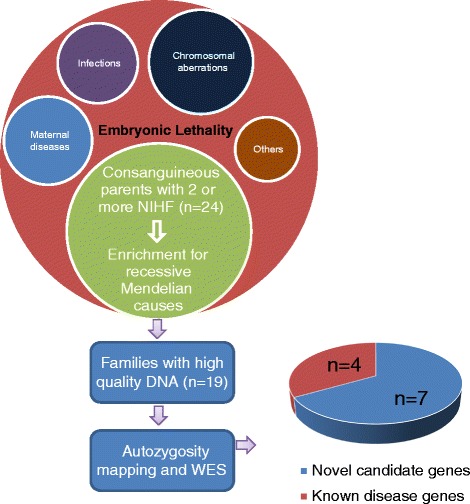
Workflow of the study

We have identified 24 consanguineous families in which two or more pregnancies were diagnosed with lethal non-immune hydrops fetalis (NIHF; Figure S1 in Additional file 1). High quality DNA was available from at least one affected fetus in 19 of the 24 families and these were subjected to whole-exome sequencing (WES). Additional file 2 summarizes the raw data characteristics from these 50× coverage exomes. This was followed by gene discovery using the algorithm outlined in Fig. 1 and detailed in Additional file 3. In seven of these families, WES revealed a likely pathogenic variant in a gene known to cause NIHF in severe cases (Table 1). Family 13DG1635 had a homozygous truncating mutation in *CTSA*, which predicts galactosialidosis, a known cause of NIHF [4–6]. Similarly, families 14DG1727, 14DG1819, 13DG0042, 13DG2155, and 14DG0947 had likely pathogenic mutations in *GUSB* as the only variants that remained after applying the various filters. These mutations predict *GUSB*-related mucopolysaccharidosis VII, another established cause of NIHF in severe cases [7, 8]. Family 13DG0975 was found to harbor a homozygous stopgain mutation in *NEB* (c.21076C > T:p.R7026X), which predicts a severe form of nemaline myopathy, which has rarely been reported to cause NIHF [9]. Family 10DG0827 was also found to harbor a homozygous mutation in the muscle-related gene *CHRNA1* (c.762C > T:p.R254C) and we reported this family as the first application of WES to identify the cause of recurrent fetal loss in humans [10]. The candidate causal variants in the remaining families, on the other hand, involve genes that have not been previously linked to human phenotypes, thus highlighting them as potential candidates for embryonic lethality.

**Table 1:** Summary of the variants identified by WES of families with recurrent fetal loss

Patient ID	Variant remaining after WES filtering	Effect of the mutation	Relevance to embryonic lethality
14DG1727	*GUSB*:NM_000181:exon2:c.307C > T:p.R103W	Replaces a highly conserved amino acid (PolyPhen score 1; SIFT score 0)	Known cause of NIHF in severe cases
14DG1819	*GUSB*:NM_000181:exon10:c.1586A > G:p.Y529C	Replaces a highly conserved amino acid (PolyPhen score 1; SIFT score 0)	Known cause of NIHF in severe cases
10DG0827	*CHRNA1*:NM_001039523:exon7:c.762C > T:p.R254C	Replaces a highly conserved amino acid (PolyPhen score 1; SIFT score 0)	Known cause of NIHF in severe cases
09DG01201	*PIGC*:NM_002642:exon2:c.659 T > C:p.L220P	Replaces a highly conserved amino acid (PolyPhen score 0.998; SIFT score 0.01)	Novel candidate
12DG2262	*UBN1*:NM_016936:exon14:c.2356 T > A:p.L786M	Replaces a highly conserved amino acid (PolyPhen score 0.915; SIFT score 0.02)	Novel candidate
13DG0042	*GUSB*:NM_000181:exon7:c.1144C > T:p.R382C	Replaces a highly conserved amino acid (PolyPhen score 0.995; SIFT score 0)	Known cause of NIHF in severe cases
13DG0259	*None*	-	-
13DG0447	*DNAH14*:NM_001373:exon23:c.3755 T > A:p.M1252K	Replaces a highly conserved amino acid (PolyPhen score 0.805; SIFT score 0)	Novel candidate
13DG0806	*THSD1*:NM_018676:exon3:c.617G > A:p.C206Y	Replaces a highly conserved amino acid (PolyPhen score 0.999; SIFT score 0)	Novel candidate
13DG0975	*NEB*:NM_001164507:exon140:c.21076C > T:p.R7026X	Truncation of >50 % of the protein	Known cause of NIHF in severe cases
13DG1635	*CTSA*:NM_000308:exon6:c.649delC:p.L217fs	Truncation of >75 % of the protein sequence	Known cause of NIHF in severe cases
13DG1885	*MYOM1*:NM_003803:exon38:c.4987G > A:p.V1663M	Replaces a highly conserved amino acid (PolyPhen score 0.999; SIFT score 0)	Novel candidate
13DG2155	*GUSB*:NM_000181:exon3:c.398G > C:p.W133S,	Replaces a highly conserved amino acid (PolyPhen score 0.984; SIFT score 0)	Known cause of NIHF in severe cases
14DG0052	*FZD6*:NM_001164616:exon5:c.773A > G:p.Y258C	Replaces a highly conserved amino acid (PolyPhen score 1; SIFT score 0)	Novel candidate
14DG0946	*THSD1*:NM_018676:exon3:c.617G > A:p.C206Y	Replaces a highly conserved amino acid (PolyPhen score 0.999; SIFT score 0)	Novel candidate
14DG0947	*GUSB*:NM_000181:exon7:c.1069C > T:p.R357X	Truncation of >75 % of the protein sequence	Known cause of NIHF in severe cases
14DG1037	*GALNT14*:NM_024572:exon13:c.C1273T:p.R425X	Truncation of >75 % of the protein sequence	Novel candidate
14DG1695	*THSD1*:NM_018676:exon3:c.617G > A:p.C206Y	Replaces a highly conserved amino acid (PolyPhen score 0.999; SIFT score 0)	Novel candidate
14DG11738	*THSD1*:NM_018676:exon3:c.G670A:p.R224X	Truncation of >75 % of the protein sequence	Novel candidate

**Fig. 2:**
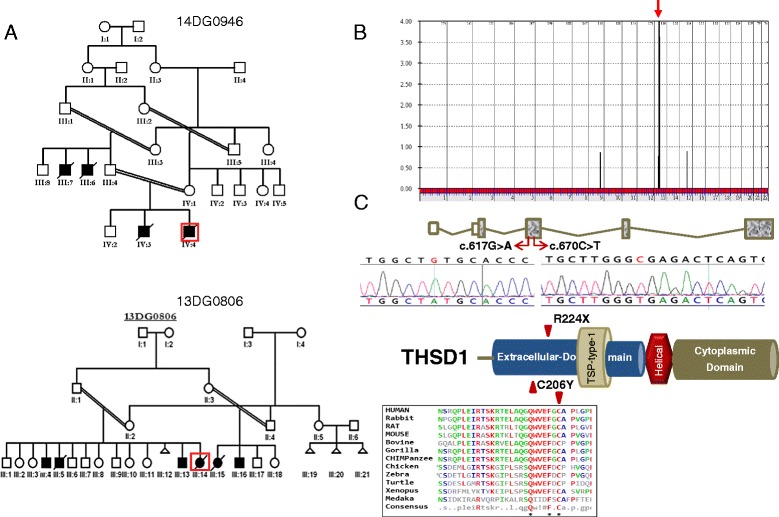
**a** Pedigrees of the two families that generated linkage to *THSD1* (index boxed in red). **b** Genome-wide linkage analysis showing a single significant peak (*red arrow*) that spans the *THSD1* locus. **c** Cartoon of the *THSD1* gene and protein with the location of the two identified mutations marked. The missense mutation replaces a highly conserved amino acid across species

The single candidate variant that survived the various filters in two of the remaining families was identical — a missense mutation in *THSD1* (c.617G > A:p.C206Y) — and linkage analysis combining the two families revealed a single linkage peak that overlaps with *THSD1* (Fig. 2). Surprisingly, in family 13DG0806, the missense mutation in *THSD1* was also found in the homozygous state in two siblings who are said to have had severe edema on prenatal ultrasound and on postnatal examination but that gradually resolved with age. Since this suggests that *THSD1* mutation can be compatible with an attenuated phenotype, we decided to enroll two consanguineous families in which NIHF was not associated with a lethal outcome. In family 14DG1695, the mother presented during pregnancy and her fetus had typical findings of NIHF but gave history of two previous children with a similar presentation who later improved spontaneously. Sequencing of *THSD1* in the fetus confirmed the presence of the same missense mutation. Unfortunately, this fetus died shortly after birth due to severe edema with respiratory compromise. Family 14DG1738 is another consanguineous family in which two siblings presented with persistent lymphedema and have history of NIHF during their pregnancies. Sequencing of *THSD1* revealed a homozygous truncating mutation (c.G670A:p.R224X; Fig. 2; Figure S2 in Additional file 1). This variant has been reported to be heterozygous in two individuals in the ExAC browser (rs9536062) for an allele frequency of 0.00001648, which is compatible with this being a disease-causing allele. Reassuringly, WES in these two families independently revealed the same mutations by applying the filters we used in the other study families.

In addition to *THSD1*, the candidate genes identified in the remaining families have not been previously linked to human diseases and thus represent novel candidates for embryonic lethality (*PIGC*, *UBN1*, *DNAH14*, *MYOM1*, *FZD6*, and *GALNT14*).

## Discussion

The spectrum of phenotypes associated with Mendelian disorders is very wide, and embryonic lethality can be viewed as the severe end of that spectrum [1]. It has long been noted that embryonic lethality can be the presenting feature of known Mendelian disorders in severe cases. However, until recently it has not been possible to study embryonic lethality as the “entry” phenotype because the phenotype is usually too nonspecific to guide testing. WES represents an exciting methodology that bypasses this limitation since it targets the entire coding genome regardless of the phenotype [10]. In this study, we extended our experience in combining the power of WES with autozygosity mapping for determining the genetic causes of many recessive Mendelian conditions in our highly consanguineous population [11] to the study of embryonic lethality as another recessive Mendelian condition in selected families. Specifically, we targeted multiplex consanguineous families in which embryonic lethality likely follows a recessive pattern. Our choice of lethal NIHF was based on the longstanding experience with this phenotype as the final common pathway of diverse pathophysiological perturbations leading to fetal loss, many of which are genetic in etiology, especially in recurrent cases [12].

The mutations we identified in *GUSB*, *CTSA*, *NEB* and *CHRNA1* can be viewed as “positive controls” in that they lend credence to the methodology used in this study to identify the cause of embryonic lethality. These genes are known to be mutated in established Mendelian diseases (mucopolysaccharidosis, galactosialidosis, nemaline myopathy and fetal akinesia, respectively) that present as embryonic lethality in severe cases. On the other hand, the genes identified in the other families have not been previously linked to any Mendelian phenotype so they represent novel candidate genes. In the case of *THSD1*, it appears that mutations in this gene cause a range of Mendelian phenotypes ranging from a lethal form of NIHF to self-limited lymphedema that resolves with age. For the other novel candidate genes, their identification in one family each precludes speculation on the range of phenotypes they may cause besides the proposed link to embryonic lethality.

*THSD1* is the most compelling candidate we identified in this study in view of the linkage data and the identification of two independent mutations. It encodes a thrombospondin type 1 domain-containing protein of poorly understood function. It was first identified in 2006 as a marker of primitive hematopoietic stem cells and endothelial cells [13]. Recently, it has been proposed that THSD1 has a potential role in angiogenesis and maintenance of vascular integrity [14]. Thus, it is tempting to speculate that the mutations we identified in this gene compromise vascular integrity resulting in a range of phenotypes from embryonic lethality to persistent or self-limiting edema as we observed in the families we report in this study.

Unlike *THSD1*, however, the remaining novel genes remain candidates since they were observed to be mutated only once, and additional families will be required to confirm their proposed link to embryonic lethality. Nevertheless, available information on the function of these genes and their involvement in basic cellular functions appear suggestive. For example, *PIGC* encodes an endoplasmic reticulum membrane protein required for the first step of glycosylphosphatidylinositol (GPI) biosynthesis [15]. GPI is a major posttranslational modification of many eukaryotic proteins to anchor them to membranes. Disruption of GPI2 (the yeast homolog of *PIGC* in human) is lethal in yeasts [16]. Similarly, *GALNT14* encodes an enzyme that catalyzes the first step in the O-glycosylation of mammalian proteins by transferring N-acetyl-D-galactosamine (GalNAc) to peptide substrates. The homozygous truncating mutation we identified in this gene will likely cause a severe form of O-glycosylation disorder that may have resulted in embryonic lethality in the respective family. It is worth highlighting that disruption of the genes involved in this pathway are lethal in yeast and mouse [17, 18]. Available literature supports the candidacy of the other candidate genes as well. *UBN1* encodes an essential component of a chromatin remodeling complex that also includes HIRA [19]. Intact UBN1 is required for HIRA stabilization and proper localization, and *HIRA*^−/−^ is embryonic lethal in mouse [20, 21]. *MYOM1* encodes myomesin, a major binding partner of another sarcomeric protein Titin, a major protein component of striated muscles [22]. Homozygous deletion of the Titin domain that interacts with myomesin results in embryonic lethality in mouse [23]. For the remaining genes, there is insufficient evidence to support their potential role in embryonic lethality.

## Conclusions

Our study, to our knowledge, represents the first attempt to systematically catalogue monogenic causes of embryonic lethality in humans by combining WES and autozygome analysis in selected families. The apparently high yield of this approach in the study cohort justifies future expansion to hundreds of similarly selected families to accelerate the discovery of embryonic lethal genes in humans.

## Materials and methods

### Human subjects

Families were enrolled in this study if parents were consanguineous and had a history of at least two pregnancies diagnosed with lethal forms of NIHF using standard criteria used in maternal-fetal medicine. After signing written informed consent, as part of an institutional review board-approved research protocol in compliance with the Declaration of Helsinki (KFSHRC RAC# 2080006 and 2121053), a blood sample from at least one affected fetus was obtained from the umbilical cord and stored in EDTA tubes for further analysis. Detailed morphological examination by ultrasonography and, when applicable, postnatal clinical examination were recorded.

### Autozygosity mapping

Determination of the autozygous genomic intervals (autozygome) was achieved by using runs of homozygosity (ROH) that are >2 Mb in size extracted from a genome-wide genotyping array as described before [24]. Briefly, DNA extracted from whole blood was run on Axiom SNP Chip platform following the manufacturer’s instructions (Affymetrix) followed by ROH determination using AutoSNPa software [25].

### Whole-exome sequencing

Exome capture was performed using TruSeq Exome Enrichment kit (Illumina) following the manufacturer’s protocol. Samples were prepared as an Illumina sequencing library, and in the second step, the sequencing libraries were enriched for the desired target using the Illumina Exome Enrichment protocol. The captured libraries were sequenced using an Illumina HiSeq 2000 Sequencer. The reads were mapped against UCSC hg19 [26] by BWA [27]. The SNPs and Indels were detected by SAMTOOLS [28].

### Mapping of likely causal variants

Variants from WES were filtered such that only novel (or very low frequency, 0.1 %), coding/splicing, homozygous variants that are within the autozygome of the affected fetus and are predicted to be pathogenic were considered as likely causal variants [29]. Frequency of variants was determined using publically available variant databases (1000 Genomes, Exome Variant Server and ExAC) as well as a database of 560 in-house ethnically matched exomes. Pathogenicity was likely if the mutation is loss-of-function (splicing/truncating) or, in the case of missense/in-frame indels, removes a highly conserved amino acid and is predicted to be pathogenic by the two in silico prediction modules PolyPhen and SIFT. All exomes described in this study can be accessed at [30].

### Availability of supporting data

All novel variants in this study have been deposited in ClinVar with the following accession numbers: SCV000222665–SCV000222684. Access to the whole-exome data used in this study is provided through the TraBioS Data Warehouse [30].

## Additional files

Additional file 1: Figure S1. Pedigrees of the study families. **Figure S2.** The two siblings with a homozygous truncating *THSD1* mutation who presented with NIHF during pregnancy and had persistent lymphedema postnatally. Additional file 2: Table S1. Summary of the raw data from whole-exome sequencing. Additional file 3: Table S2. Details of the filters used to analyze whole-exome data.

## Abbreviations

GPI: glycosylphosphatidylinositol
NIHF: non-immune hydrops fetalis
ROH: runs of homozygosity
WES: whole-exome sequencing
