# Supplementary figures and images for: Identification of embryonic lethal genes in humans by autozygosity mapping and exome sequencing in consanguineous families
Source: Genome Biol. 2015 Jun 3;16(1):116. doi: 10.1186/s13059-015-0681-6 (PMC4491988; doi:10.1186/s13059-015-0681-6)

Figure S1a

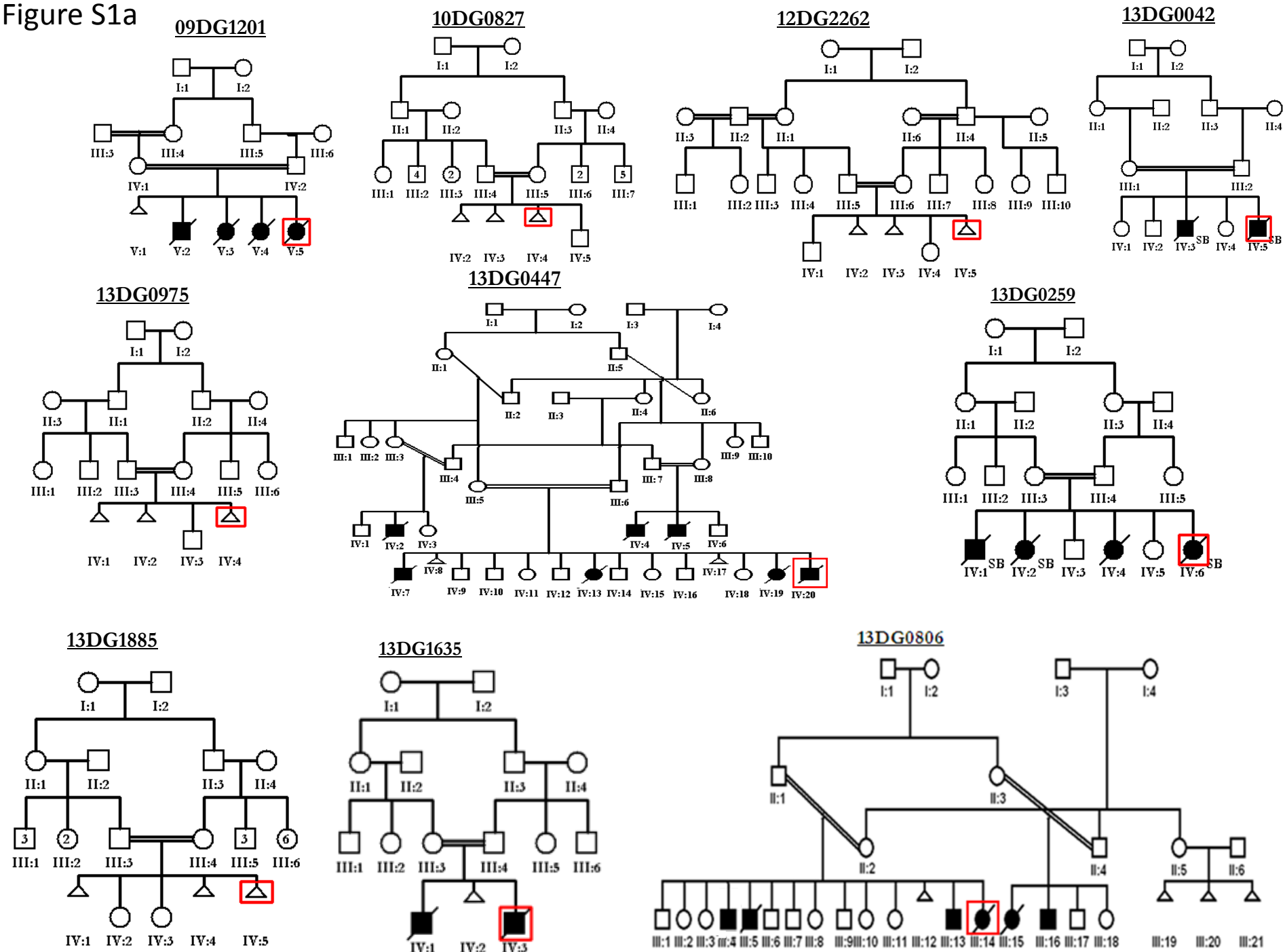

Figure S1b

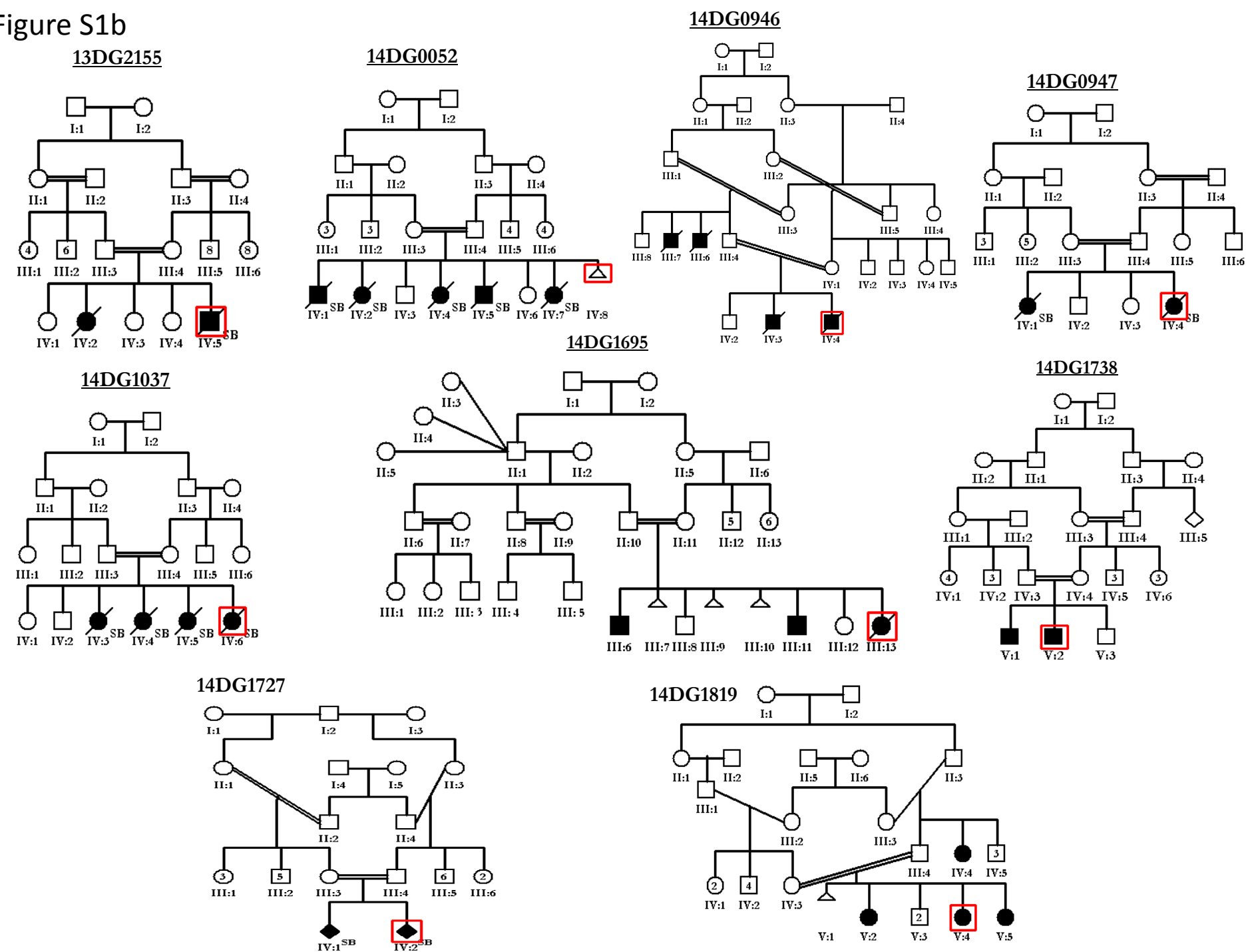

Figure S2

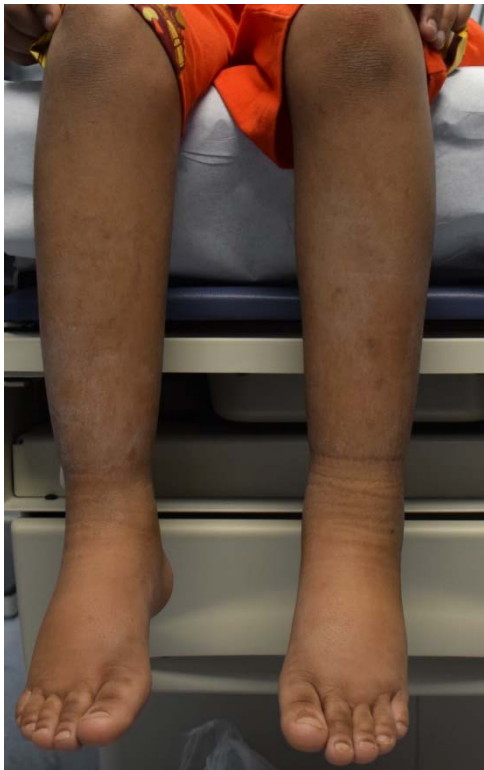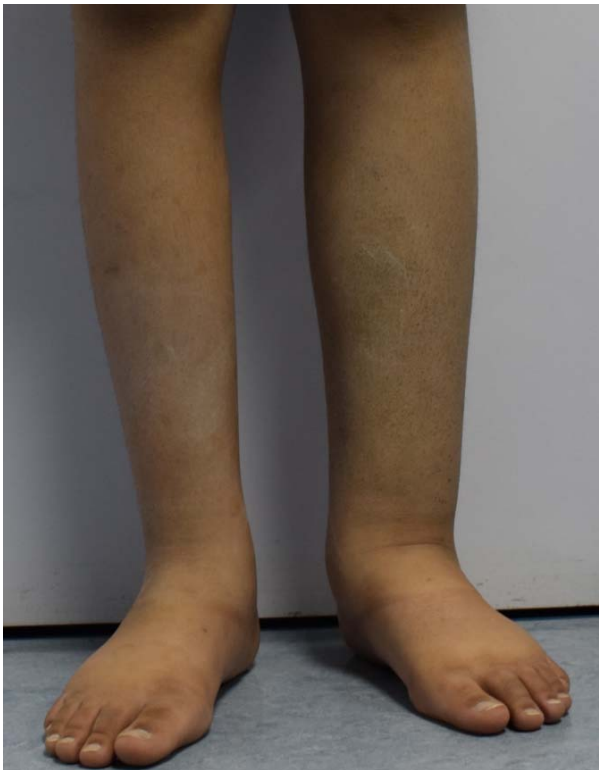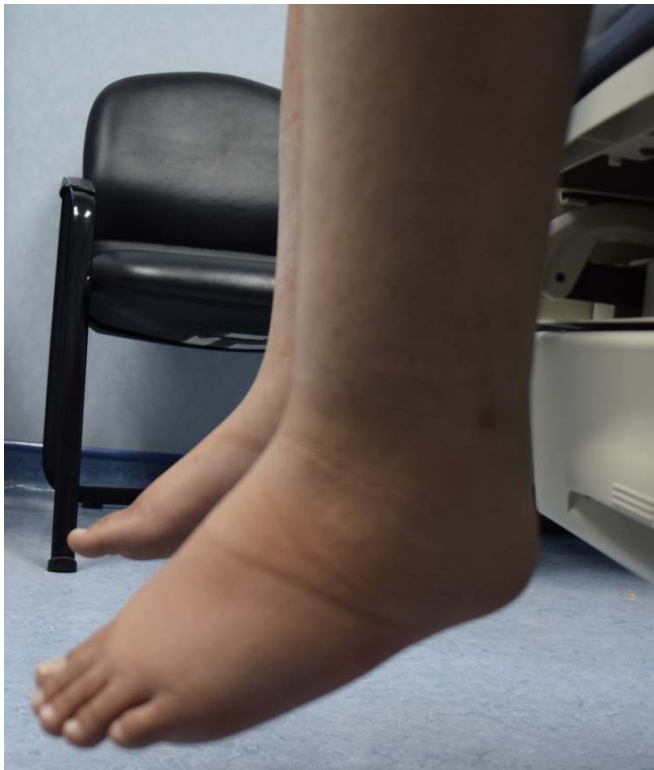

Supplement: Additional file 1: Figure S1. — Pedigrees of the study families. Figure S2. The two siblings with a homozygous truncating THSD1 mutation who presented with NIHF during pregnancy and had persistent lymphedema postnatally. [file 13059_2015_681_MOESM1_ESM.pdf]
